# Musculoskeletal anatomy: evaluation and comparison of common teaching and learning modalities

**DOI:** 10.1038/s41598-020-80860-7

**Published:** 2021-01-15

**Authors:** Aristeidis Zibis, Vasileios Mitrousias, Sokratis Varitimidis, Vasileios Raoulis, Apostolos Fyllos, Dimitrios Arvanitis

**Affiliations:** 1 Department of Anatomy, Faculty of Medicine, University of Thessaly, Panepistimion 3st Biopolis, 41110 Larissa, Greece; 2 Department of Orthopaedic Surgery, Faculty of Medicine, University of Thessaly, 41110 Larissa, Greece

**Keywords:** Anatomy, Musculoskeletal system

## Abstract

Anatomy teaching has traditionally been based on dissection. However, alternative teaching modalities constantly emerge, the use of which along with a decrease in teaching hours has brought the anatomy knowledge of students and young doctors into question. In this way, the goal of the present study is to a. compare the efficacy of the most common teaching modalities and b. investigate students’ perceptions on each modality. In total, 313 medical students were taught gross anatomy of the upper limb, using four different learning modalities: dissection (n = 80), prosections (n = 77), plastic models (n = 84) and 3D anatomy software (n = 72). Students’ knowledge was examined by 100 multiple-choice and tag questions followed by an evaluation questionnaire. Regarding performance, the dissection and the 3D group outperformed the prosection and the plastic models group in total and multiple-choice questions. The performance of the 3D group in tag questions was also statistically significantly higher compared to the other three groups. In the evaluation questionnaire, dissection outperformed the rest three modalities in questions assessing students’ satisfaction, but also fear or stress before the laboratory. Moreover, dissection and 3D software were considered more useful when preparing for clinical activities. In conclusion, dissection remains first in students’ preferences and achieves higher knowledge acquisition. Contemporary, 3D anatomy software are considered equally important when preparing for clinical activities and mainly favor spatial knowledge acquisition. Prosections could be a valuable alternative when dissection is unavailable due to limited time or shortage of cadavers. Plastic models are less effective in knowledge acquisition but could be valuable when preparing for cadaveric laboratories. In conclusion, the targeted use of each learning modality is essential for a modern medical curriculum.

## Introduction

Anatomy has had a glorious past. Glorious because it laid the foundations for all biomedical sciences, established the linguistic basis for medicine, and contributed to questioning of dogmatic principles^1^. Today, anatomy remains the cornerstone of medical education. However, the changing educational environment and the recent technological advances alter the way anatomy is taught^2^. Dissection is interwoven with anatomy teaching, since the time that Andreas Vesalius (1514–1564 AD) and William Harvey (1578–1657 AD) performed their own dissections, setting the foundations of modern anatomy and physiology^1^. Nevertheless, decreased total and laboratory hours in gross anatomy along with the shortage of cadavers have led to the use of other, often less time-consuming modalities^3–7^. Prosections are more and more used to save teaching time, although their preparation demands both time and qualified staff^8^. Plastic models are also a popular educational modality. Easy to use, low-cost and with no need for maintaining facilities, such models improve anatomy knowledge, acting as memory aids and resembling the true dimensions of the human body^9,10^. Lastly, many three-dimensional (3D) anatomy applications have become available in the last few years. Their use aroused students’ enthusiasm and participation, and their continuous development turned “virtual dissection” into a daily-routine activity^11^.

Despite the development of all the above teaching modalities, anatomy knowledge in medical students and young doctors is considered insufficient^12–16^. Studies have revealed that 57% of the directors of medical programs in the United States of America (U.S.A.) believe that their residents need to revise their anatomical knowledge^12^. Similarly, studies in the United Kingdom (U.K.) showed that 87.4% of the consultants believe that the level of anatomy knowledge is below average in medical students and 71.8% believe the same for medical trainees^15^. Interestingly, the same opinion is also expressed by students. In a U.K. study, the majority of students were dissatisfied with their anatomical knowledge, at the beginning of their medical career^17^. Also, in the study of Fitzgerald et al., 50% of young doctors think that the anatomy they were taught is insufficient^18^. Even in studies where residents believe that their course in gross anatomy prepared them well for clinical practice, program directors rate these residents less prepared than the residents rated themselves^16^. Considering that in the U.K. there was a sevenfold increase in claims associated with anatomical errors submitted to the Medical Defense Union and that some out of 80,000 avoidable deaths in the U.S. could be attributed to anatomical incompetence, ameliorating anatomy education is crucial^13,19,20^.

Evidence-based teaching in musculoskeletal anatomy is still rudimentary since studies investigating how each teaching modality affects performance are missing. In this way, the goal of this study is to investigate (a) whether there is any difference in effectiveness between dissection, prosections, plastic models and 3D anatomy software, as assessed by students’ performance in examinations and (b) whether there is any difference in students’ perceptions of each teaching modality, based on their answers in the evaluation questionnaire. To our knowledge, this is the first study comparing these four modalities simultaneously, when used in teaching musculoskeletal anatomy. The null hypothesis is that there is no significant difference between the four modalities neither in students’ performance in the examinations nor in students’ perceptions.

## Materials and methods

### Time and recruitment

The study took place between 2014 and 2019. Volunteer, first-year, undergraduate medical students were recruited for the needs of this study. Previous anatomy knowledge was the only exclusion criterion. A pre-test was not necessary since anatomy is not taught during secondary education in the country where the study took place. However, students holding a past degree or reporting participation in relevant educational activities (e.g. previous degree in health sciences, anatomy summer schools) were excluded from the study to ensure that the level of anatomy knowledge was the same for all participants at the beginning of the study. The educational process was designed to take place during the first semester of each year, before formal anatomy teaching, since in the university’s formal curriculum anatomy is part of the second semester.

Each year, approximately 15 days before the scheduled start of the educational activities, a promotional presentation was held to inform students about the goals of the study, as well as the benefits and the obligations of participating in it. All potential participants were informed about the duration and the location of the study, however, details on the teaching modality and the teaching subject were concealed to prevent a possible foreknowledge bias. Investigators were blinded to the identity of the participants and students were informed that both the examination sheet and the evaluation questionnaire would be anonymous, to ensure unbiased answers and reduce performance stress.

Ethical approval for the research was obtained by the Institutional Review Board of the University of Thessaly, Greece (Reference number: 848). This research was performed in accordance with the Declaration of Helsinki Ethical Principles for Medical Research involving Human Subjects and an informed consent form was also signed by all participants.

### Sample, study design and materials

The study was designed to compare students’ performance and perceptions following exposure to musculoskeletal anatomy of the upper limb, by using four different teaching modalities. Every year, four randomized groups of students were created, using the auto-draw computer program RandomPicker (version 4.0, www.randompicker.com, Veromotion, s.r.o., Prague, Czech Republic). Group 1 used lectures and dissection, group 2 used lectures and prosections, group 3 used lectures and plastic models and group 4 used lectures and 3D software. The complete sample of the study was the sum of the four consecutive annual samples, which are presented in Table 1.

**Table 1:** Total sample and participants’ distribution in each group yearly.

Modality	2014	2015	2016	2017	Total
Lectures and dissection	21	22	23	21	87 (34♂:53♀)
Lectures and prosections	20	23	23	21	87 (36♂:51♀)
Lectures and 3D software	20	22	24	21	88 (38♂:50♀)
Lectures and plastic models	20	22	23	22	87 (36♂:51♀)
Total	81	89	93	85	349 (144♂:205♀)

**Figure 1:**
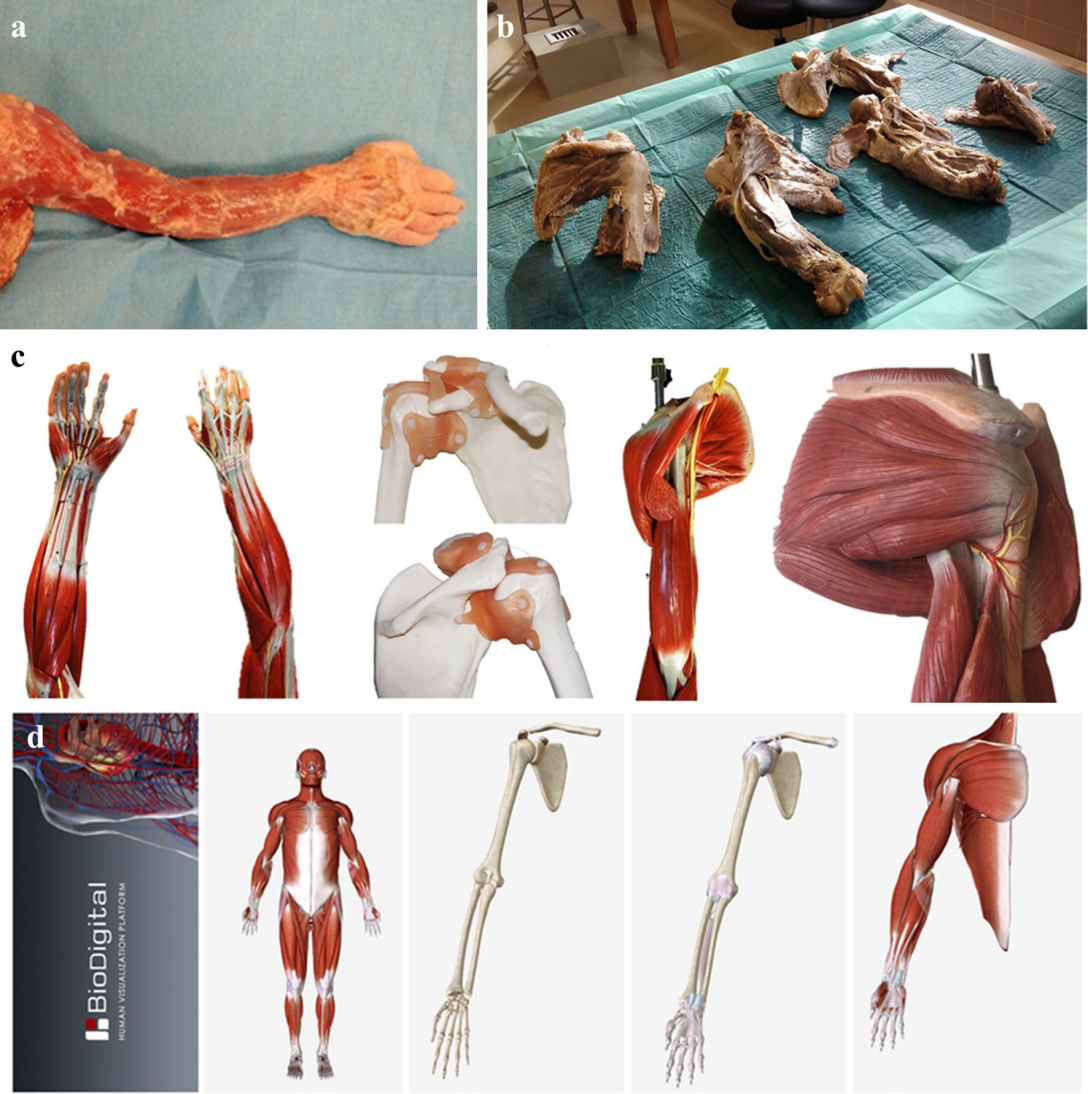
Examples of the fresh-frozen cadaveric specimens (**a**), the prosections (**b**), the plastic models (**c**) and views of the BioDigital Human, 3D anatomy software (**d**), which were used during laboratories.

The upper limb was chosen since it was perceived as the second most complex region following head and neck, in musculoskeletal anatomy education, by first-year medical students of the department of anatomy, of our university^21^. Dissection was performed on four fresh-frozen cadaveric upper limbs, available in the department of anatomy. Similarly, four prosections were prepared by one anatomist and one Ph.D. student, using cadaveric material provided by the department of anatomy. Each specimen was appropriately pre-dissected, in order to be used for demonstration of different anatomical structures and layers and preserved in formalin solution. For example, four different cadaveric shoulder specimens were suitably pre-dissected to demonstrate (a) muscles of the superficial layer, (b) muscles of the rotator cuff, (c) ligaments and bony structures. Plastic models (SOMSO Modelle) were already available in our department. In the same way, different models were available for different layers and different anatomical structures (bones, ligaments, deep muscles and superficial muscles). The software used was the BioDigital Human (version 1.0.4, www.biodigital.com, New York, USA), which is a freeware, rated 4 + and available in Windows, Android and iOS. The BioDigital Human was installed in the computer room of the department of anatomy. The name of the software was concealed during the workshop. Examples of the cadaveric specimens, the plastic models and views of the BioDigital Human software used during laboratory sessions, are available in Fig. 1.

### Educational process

Each year, two lectures and two laboratories were conducted for each group, each lasted 2 h. The first educational session (1st Lecture and 1st Laboratory) was dedicated to bone and ligament anatomy, and the second one to muscle anatomy. Lectures were identical for all groups. All images used in lectures were taken by the Platzer Color Atlas and Textbook of Human Anatomy^22^, which is the book formally used by the department of anatomy in musculoskeletal anatomy teaching. Detailed handouts of both lectures were given to students to assist them during laboratory work.

At the beginning of each laboratory session, a 20-min presentation of all structures, took place by the tutor. After a 10-min break for questions, students had the chance to explore all structures on their own for 90 min. In group 1, cadaveric, upper limb specimens were dissected. Basic surgical instruments (scalpel, forceps, scissors) were used by all students. As 90 min for muscular dissection of the upper limb is limited enough, the skin and subcutaneous tissue had been already removed. In addition, the instruction to the students was not to dissect the vessels and nerves, which they could even sacrifice during the preparation of the muscles. The introductory dissection performed by the tutor was recorded and projected on wall-mounted screens, placed in front of each dissection table, to assist novice students with a step-by-step video demonstration of the whole process. In group 2, pre-dissected upper limb specimens were used (prosections). In group 3, plastic models were used instead of cadaveric specimens. Students had the chance to assemble and disassemble the plastic models, removing superficial structures to explore the deeper ones. In group 4, the BioDigital Human software was used. Students had the chance to explore all structures using “rotate”, “zoom in/out”, “dissect” for removing structures and “isolate mode” for isolating structures. All four groups worked in small teams of 4–5 persons. Students to modality ratio was approximately 4:1. Exposure of students in each modality took place only inside the laboratory and the exposure time was exactly the same for all groups. Two days elapsed, between each session. The same frame is also used for teaching the upper limb, musculoskeletal anatomy in the university’s, formal, medical curriculum. All lectures and all laboratory sessions were accomplished and supervised by one tutor, who was the same for all groups, with more than ten years of experience in anatomy education. The same educational strategy has been also used in previous studies of our department^21,23^.

### Examination process and de-identification

Each year, approximately two days after the completion of the learning sessions, students participated in the final examinations. Attendance to all lectures and laboratories was required for participating in the examination process. One hundred questions were used to evaluate each group. Half of the questions asked students to identify anatomical structures in projected images (tag questions) and the rest half were multiple-choice questions (McQ), designed to address the first two levels of cognitive objectives in Bloom’s taxonomy (knowledge and comprehension)^24^ and assessing muscle origin, insertion, and nerve supply (Bloom’s level 1–25 questions, Bloom’s level 2–25 questions). Both tag and McQ were identical for all four groups. In tag questions, two kinds of images were used: cadaveric images and anatomy atlas’ images. Namely students from all four groups were asked to identify structures both in cadaveric images and in atlas’ images. Each image was being projected for 30 s. McQ were selected from the collection of previous examination subjects and the level of difficulty was as high as in the formal, medical school examinations. All McQ have been created and reviewed by the department’s tutors and Ph.D. students, following recent recommendations and the blooming anatomy tool to ensure appropriateness, avoid cues, and enhance reliability^25,26^. The whole examination process lasted 100 min (30 s for each tag question and 90 s for each McQ). Tag and McQ examples are provided in the "Appendix".

To prevent cheating, the examination process took place in an auditorium and was strictly supervised. Examinations were anonymous. Α six-digit code was chosen and written on the answer sheet by each student, instead of his/her name. All data were collected and analyzed using this specific code, which was strictly personal. Each year, after the end of the examination process, results corresponding to each code were announced. A similar examination protocol has been used in previous studies accomplished by our department^21,23^.

### Questionnaire and evaluation of the teaching modalities

After the examinations, students were asked to fill in an anonymous questionnaire to evaluate the modality they used. The questionnaire was developed by the investigators based on current literature. Two Ph.D. students, one anatomist and two orthopaedic surgeons with expertise in anatomy teaching reviewed the questionnaire for clarity and face validity. The questionnaire consisted of 10 questions. In the first part, two questions were used to collect demographical data (sex, age). The second part consisted of six, 5-point, unipolar Likert-scales (four for assessing the teaching modality and two investigating students’ feelings). In the third part, there were two questions: a 5-point, unipolar Likert-scale investigating participants’ intention to suggest the course to the forthcoming students and an open-ended question asking students for a brief comment and to state what they would like to be different/to be added in the educational process. The same six-digit code used in the examination sheet was also used in the questionnaire by each student, to match the examination sheets with the evaluation form. All questions are available in the "Appendix". Parts of the present questionnaire have been also used in past studies conducted by our department^21,23^.

### Statistical analysis

All data were analyzed using the SPSS statistical package, version 21.0 for Windows (https://www.ibm.com/analytics/spss-statistics-software, IBM Corp., Armonk, NY, USA). Descriptive and inferential statistics were performed. The level of statistical significance was set at 0.05.

One-way ANOVA was used to compare students’ scores in the examination process. Outliers were assessed by a boxplot. Normality was checked using the Shapiro Wilk’s test. Homogeneity of variances was assessed using Levene’s test. When homogeneity of variances was met, the Tukey–Kramer post hoc test was used to assess group differences. When homogeneity of variances was violated, the Games-Howell post hoc test was used to assess group differences. The effect size was assessed using Hays’ ω2. Paired-samples t-test was used to compare students’ scores of the same group in different types of questions. Student’s t-test was used to assess differences in scores between male and female students of each group.

Students’ answers in the Likert-scale questions were compared using the Kruskal–Wallis H test. Distributions of scores were not similar for all groups, as assessed by visual inspection of a boxplot. When the Kruskal–Wallis H test was statistically significant (*p* < 0.05), all pairwise comparisons were made using Dunn's (1964) procedure with a Bonferroni adjustment. Finally, the one proportion z-test was used to assess differences in students’ proportions of the same group, feeling fear/stress before and after training in lab sessions. All expected cell frequencies were greater than five. The level of statistical significance was set at 0.05.

#### Quality of multiple-choice questions

Item analysis was used to assess the quality of McQ. Cronbach’s alpha was used to measure internal consistency and multiple-choice questions’ reliability^27,28^. Each item was analyzed for difficulty index (P) and discrimination index (D). Mean difficulty index (P) was 0.48 while mean discrimination index (D) was 0.34. More specifically, difficulty index of 91% of McQ was in the acceptable range (P = 0.3–0.7) while 9% of McQ were too difficult (P < 0.3). Discrimination index of 33% of McQ was excellent (D > 0.4), 29% were good (D = 0.3–0.39), 22% were acceptable (D = 0.2–0.29), and 16% were poor (D < 0.19). Cronbach’s alpha value, used to assess reliability, was 0.877 (good).

#### Quality of the evaluation questionnaire

Data were analyzed by exploratory factor analysis, using principal components analysis (PCA). The appropriateness of data was checked with Kaiser–Meyer–Olkin (KMO) measure, which examines correlations among the items and its values should be greater than 0.6 for a satisfactory analysis to be achieved^27,28^. The KMO value in the present study was 0.793, while Bartlett’s test chi-square was equal to 1184.217, *p* < 0.001. Of the four initial factors extracted, interpreting the 64% of the total variance, two putative factors were finally formed, according to factor loadings and conceptual coherence. The items per factor and their loadings were as follows:

Factor 1 (arbitrarily called “general perceptions”) consisted of five items, i.e. item (factor loading). (1). Satisfaction from the teaching method (0.738); (2). Anticipations of the teaching method (0.707); (3). Educational value of the teaching method (0.534); (4). Usefulness of the teaching method for future clinical skills (0.511); and (5). Propose the course to the forthcoming students (0.595).

Factor 2 (arbitrarily called “psychological aspect”): (1). Stress/discomfort before laboratory (0.895) and (2). Stress/discomfort after laboratory (0.832).

Putative factors were further analyzed for internal consistency, which is one form of reliability, using Cronbach’s alpha. Factor 1 had a Cronbach’s α value equal to 0.831 and factor 2 had a Cronbach’s α value equal to 0.733, showing satisfactory internal consistency^27,28^.

## Results

### Final sample

In total 313 students (90% of the initial sample) completed the whole course. Specifically, 80 students performed dissection (Group 1), 77 students used prosections (Group 2), 84 students used plastic models (Group 3) and 72 students used the BioDigital Human 3D anatomy software (Group 4). Sixteen students dropped out of the study. The remaining 30 students were not allowed to participate in the examinations, since they had missed either one of the lectures or one of the laboratories. Mean age of the participants was 18.4 (± 1) years old. Total sex ratio was approximately 2:3 since 43% of the participants were male and 57% were female.

### Students’ performance in the examination process (quantitative results)

#### Group-based analysis

As far as performance is considered, the null hypothesis of this study was rejected, since there was a significant difference in students’ scores between the four groups. Results of the analysis using ANOVA are presented in Table 2. The effect size was either medium or large (0.06–0.21) according to ω2 values, also presented in Table 2^29,30^.

**Table 2:** Group-based analysis using ANOVA and Tukey HSD or Games–Howell post-hoc tests.

Question type	Anova F-test *p*	Group	Ν	Mean	Standard deviation (SD)	Standard error (se)	Post hoc (Tukey HSD/Games–Howell)
Total questions	F = 16.030 *p* < 0.001 ω^2^ = 0.13	Dissection (1)	80	49.00	19.74	2.20	1 > 2,3
		Prosection (2)	77	40.98	17.48	1.99	2 > 3 and 2 < 1,4
		Plastic models (3)	84	33.59	17.50	1.90	3 < 1,2,4
		3D (4)	72	52.84	21.37	2.51	4 > 2,3
Tag questions	F = 11.561 *p* < 0.001 ω^2^ = 0.09	Dissection (1)	80	22.03	12.09	1.35	*1 < 4
		Prosection (2)	77	21.18	11.38	1.29	*2 < 4
		Plastic models (3)	84	17.14	12.47	1.36	*3 < 4
		3D (4)	72	28.80	13.78	1.62	*4 > 1,2,3
Tag questions using cadaveric images	F = 6.893 *p* < 0.001 ω^2^ = 0.06	Dissection (1)	80	10.17	5.19	0.58	No dif
		Prosection (2)	77	11.87	6.10	0.69	2 > 3
		Plastic models (3)	84	8.42	6.25	0.68	3 < 2,4
		3D (4)	72	12.36	6.61	0.78	4 > 3
Tag questions using atlas’ images	F = 19.846 *p* < 0.001 ω^2^ = 0.15	Dissection (1)	80	11.86	7.16	0.80	1 > 3 and 1 < 4
		Prosection (2)	77	9.37	5.71	0.65	2 < 4
		Plastic models (3)	84	8.71	6.63	0.72	3 < 1,4
		3D (4)	72	16.44	7.67	0.90	4 > 1,2,3
Multiple-choice questions (McQ)	F = 29.282 *p* < 0.001 ω^2^ = 0.21	Dissection (1)	80	26.95	8.61	0.96	1 > 2,3
		Prosection (2)	77	19.80	7.32	0.83	2 > 3 and 2 < 1,4
		Plastic models (3)	84	16.45	6.50	0.70	3 < 1,2,4
		3D (4)	72	24.04	8.22	0.96	4 > 2,3
Bloom 1 McQ	F = 26.981 *p* < 0.001 ω^2^ = 0.20	Dissection (1)	80	14.11	4.93	0.55	1 > 2,3
		Prosection (2)	77	10.12	4.44	0.50	2 > 3 and 2 < 1,4
		Plastic models (3)	84	8.45	3.72	0.40	3 < 1,2,4
		3D (4)	72	12.94	4.72	0.55	4 > 2,3
Bloom 2 McQ	F = 24.296 *p* < 0.001 ω^2^ = 0.18	Dissection (1)	80	12.83	4.08	0.45	*1 > 2,3,4
		Prosection (2)	77	9.67	3.51	0.40	*2 > 3 and 2 < 1
		Plastic models (3)	84	8.00	3.42	0.37	*3 < 1,2,4
		3D (4)	72	11.09	4.02	0.47	*4 > 3 and 4 < 1

*Using Tukey HSD post hoc test.

**Figure 2:**
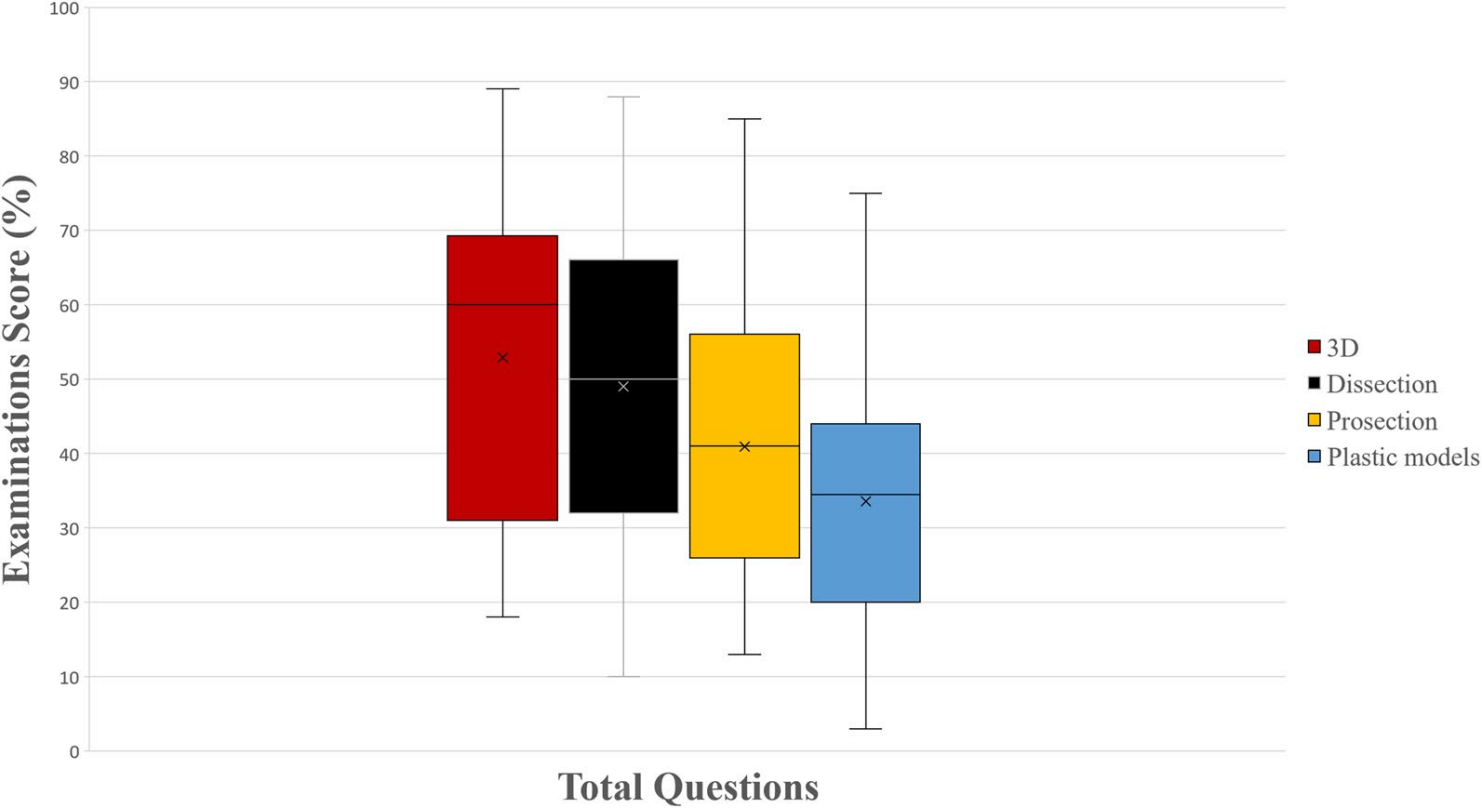
A boxplot of students’ scores (%) in the examinations, with whiskers from minimum to maximum. Performance of students in total questions is depicted. Segment inside the boxplot shows the median and “x” shows the mean. Statistically significant differences between the four groups are summarized as follows: 3D, Dissection > Prosections, Plastic Models and Prosections > Plastic Models.

In total questions, the higher performance of the 3D group (Group 4) was statistically significant compared to the prosections group (*p* = 0.002) and the plastic models group (*p* < 0.001), but there was no difference when compared to the dissection group (*p* = 0.66). The dissection group also performed statistically significantly better compared to the prosection group (*p* = 0.03) and the plastic models group (*p* < 0.001). Lastly, the prosections group performed significantly better compared to the plastic models group (*p* = 0.04). A schematic representation of students’ examination scores (%) can be seen in Fig. 2.

**Figure 3:**
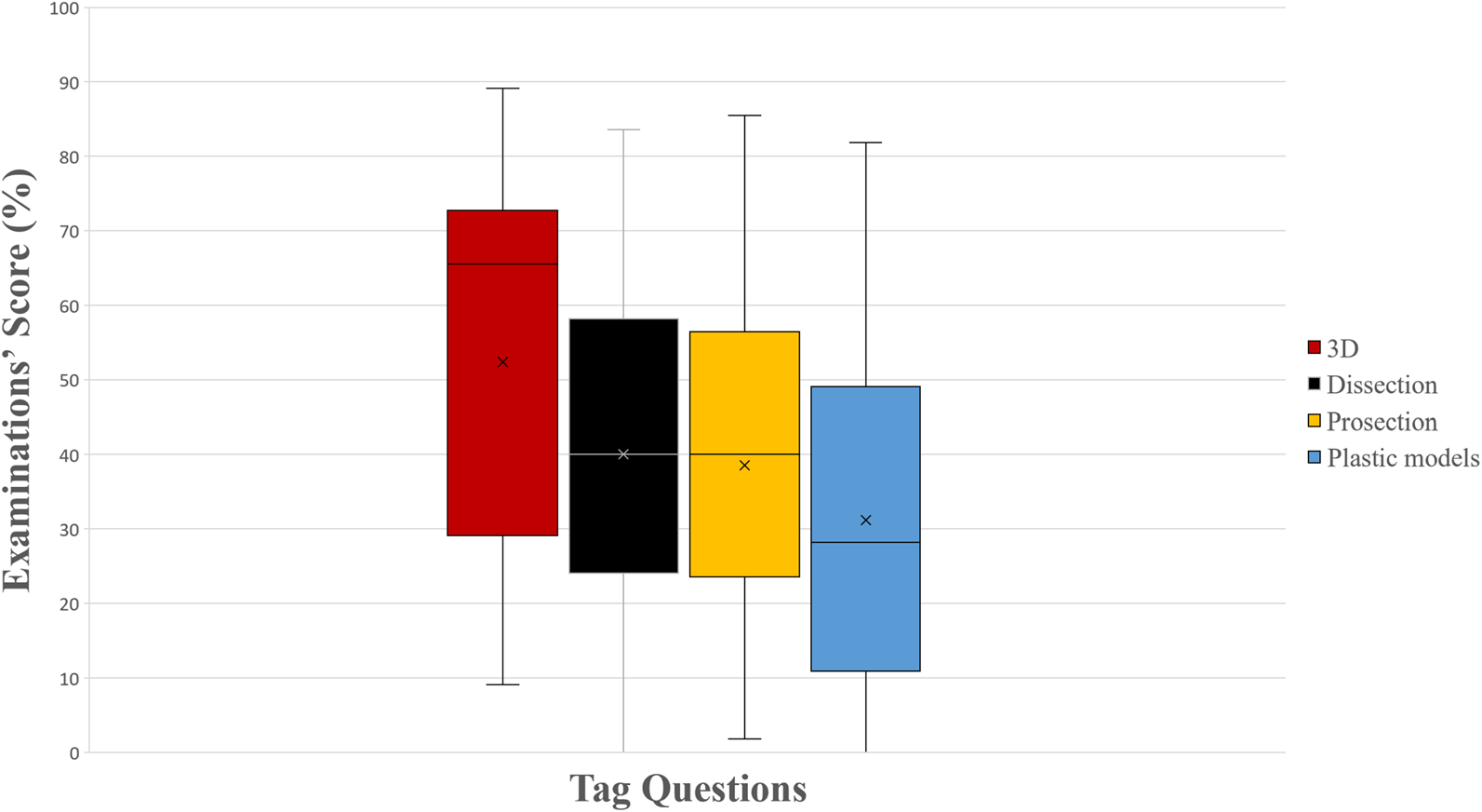
A boxplot of students’ scores (%) in the examinations, with whiskers from minimum to maximum. Performance of students in tag questions is depicted. Segment inside the boxplot shows the median and “x” shows the mean. Statistically significant differences between the four groups are summarized as follows: 3D > Dissection, Prosections, Plastic Models.

**Figure 4:**
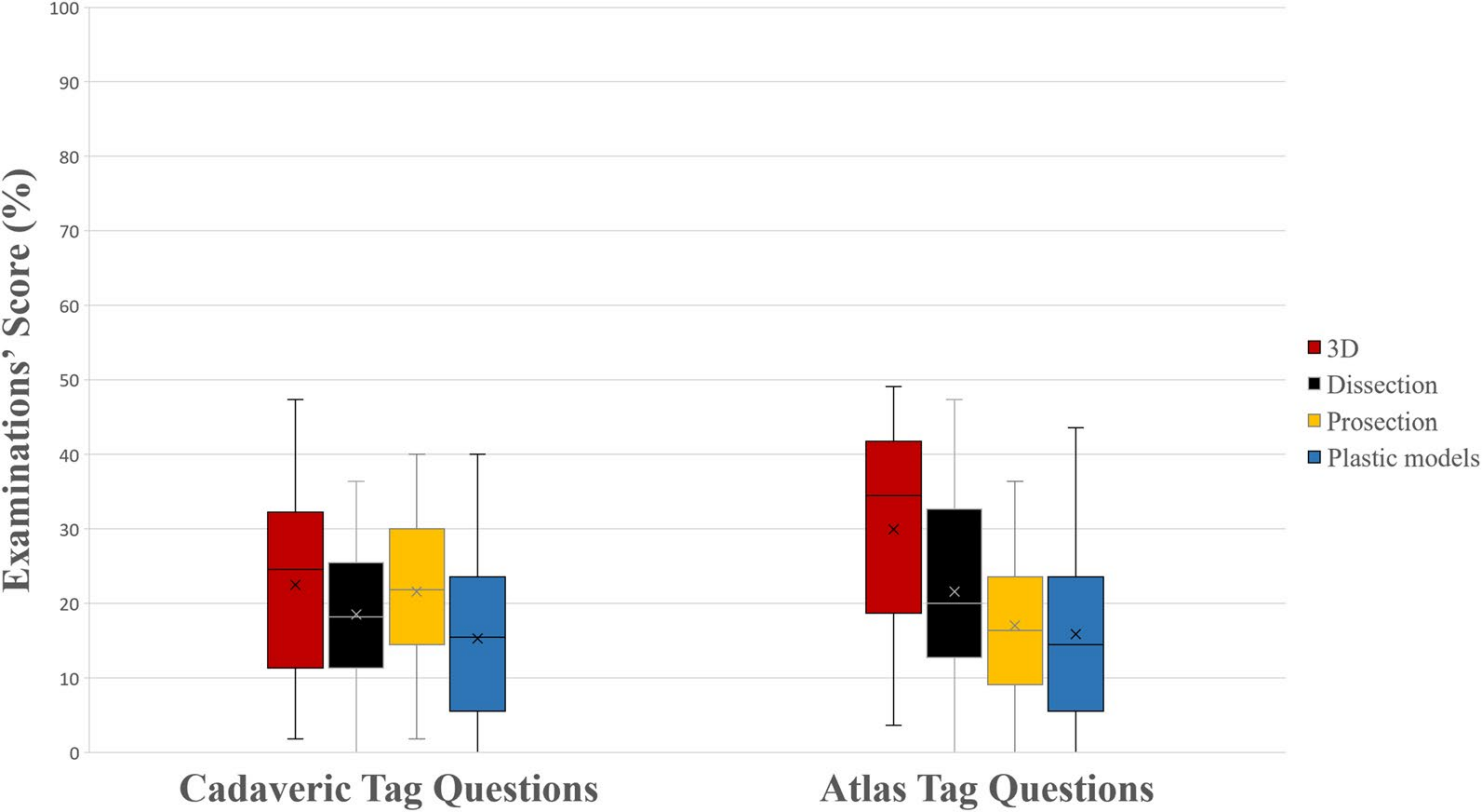
A boxplot of students’ scores (%) in the examinations, with whiskers from minimum to maximum. Performance of students in tag questions using cadaveric images and in tag questions using atlas’ images is depicted. Segment inside the boxplot shows the median and “x” shows the mean. Statistically significant differences between the four groups are summarized as follows: Cadaveric tag questions 3D, Prosections > Dissection, Plastic Models; Atlas’ tag questions 3D > Dissection, Prosections, Plastic Models and Dissection > Plastic Models.

In tag questions, the higher performance of the 3D group (Group 4) was statistically significant compared to all other groups (dissection, *p* = 0.005; prosection, *p* = 0.001; plastic models, *p* < 0.001), whose scores did not differ significantly (Fig. 3). In the sub-analysis, the 3D group also outperformed all other groups in tag questions using anatomy atlas’ images (dissection, *p* = 0.001; prosection, *p* < 0.001; plastic models, *p* < 0.001) and the dissection group outperformed the plastic models group (*p* = 0.02). In tag questions using cadaveric images, there was no statistically significant difference between the 3D group, the dissection group (*p* = 0.11) and the prosection group (*p* = 0.96). However, the 3D group and the prosections group performed significantly better compared to the plastic models group (*p* = 0.001 and *p* = 0.003 respectively), as it can be seen in Fig. 4.

**Figure 5:**
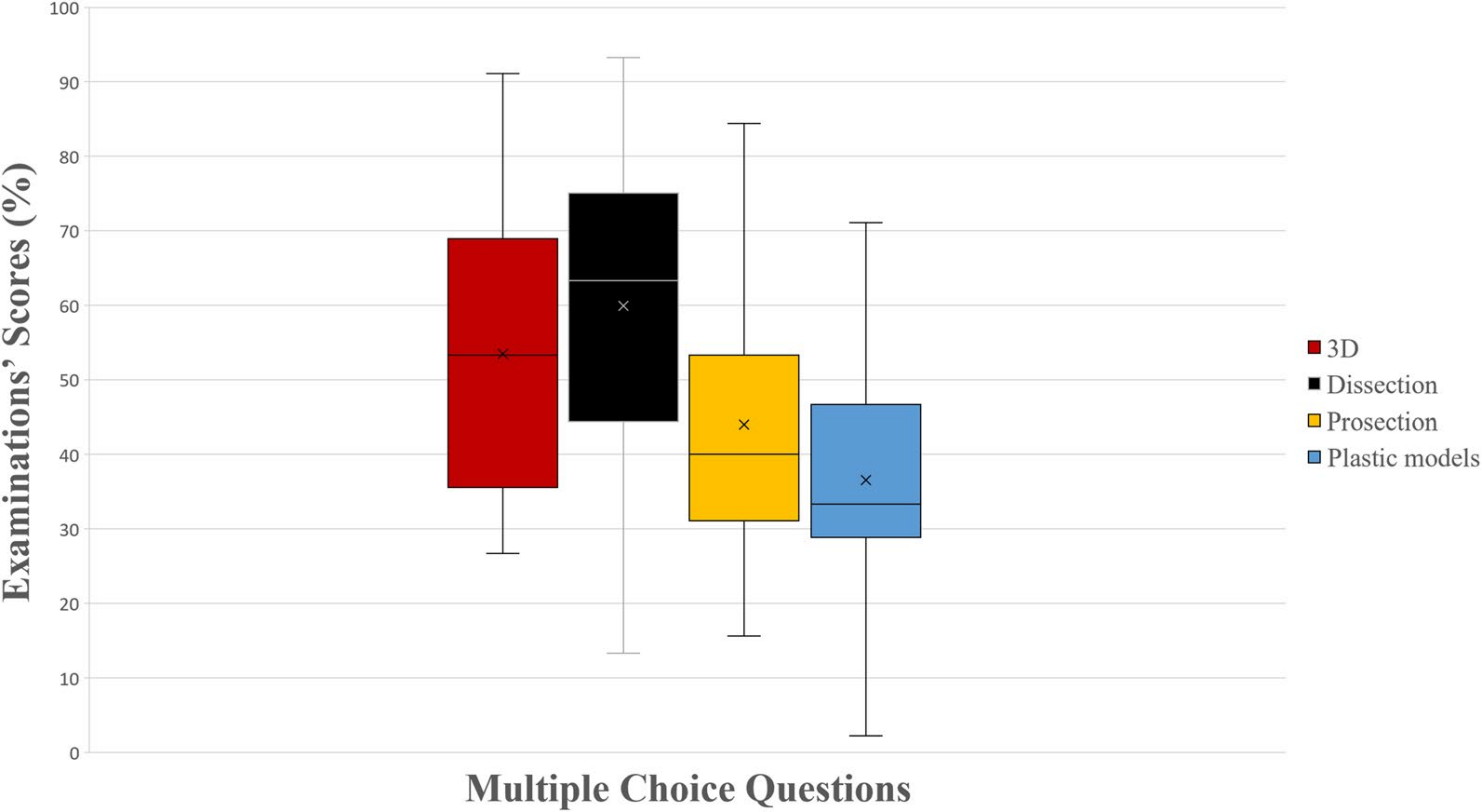
A boxplot of students’ scores (%) in the examinations, with whiskers from minimum to maximum. Performance of students in multiple-choice questions is depicted. Segment inside the boxplot shows the median and “x” shows the mean. Statistically significant differences between the four groups are summarized as follows: 3D, Dissection > Prosections, Plastic Models and Prosections > Plastic Models.

**Figure 6:**
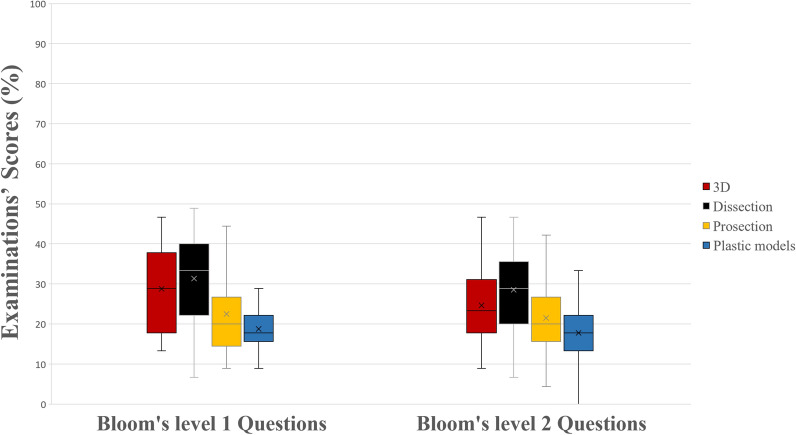
A boxplot of students’ scores (%) in the examinations, with whiskers from minimum to maximum. Performance of students in multiple-choice questions (McQ) type 1 and 2 in Bloom’ taxonomy is depicted. Segment inside the boxplot shows the median and “x” shows the mean. Statistically significant differences between the four groups are summarized as follows: Bloom 1 McQ 3D, Dissection > Prosections, Plastic Models; Bloom 2 McQ Dissection > Prosections, Plastic Models, 3D and 3D, Prosections > Plastic Models.

In McQ, similar to the total questions, the 3D group and the dissection group outperformed the prosections group (*p* = 0.006 and *p* < 0.001, respectively) and the plastic models group (*p* < 0.001 and *p* < 0.001, respectively). Students from the prosections group also performed better compared to those of the plastic models group (*p* = 0.01), as it can be seen in Fig. 5. In the sub-analysis, when examining Bloom’s type 1 questions, the dissection group and the 3D group outperformed the prosection (*p* < 0.001, *p* = 0.001) and the plastic models group (*p* < 0.001, *p* < 0.001). In Bloom’s type 2 questions, the dissection group scored significantly higher compared to the other three groups (*p* < 0.001, *p* < 0.001, and *p* = 0.02, respectively). Moreover the plastic models group performed statistically significantly lower compared to the prosections (*p* = 0.02) and the 3D group (*p* < 0.001). No other statistically significant differences were observed. A schematic representation of students’ scores (%) can be seen in Figs. 5, 6.

#### Question-based analysis

**Figure 7:**
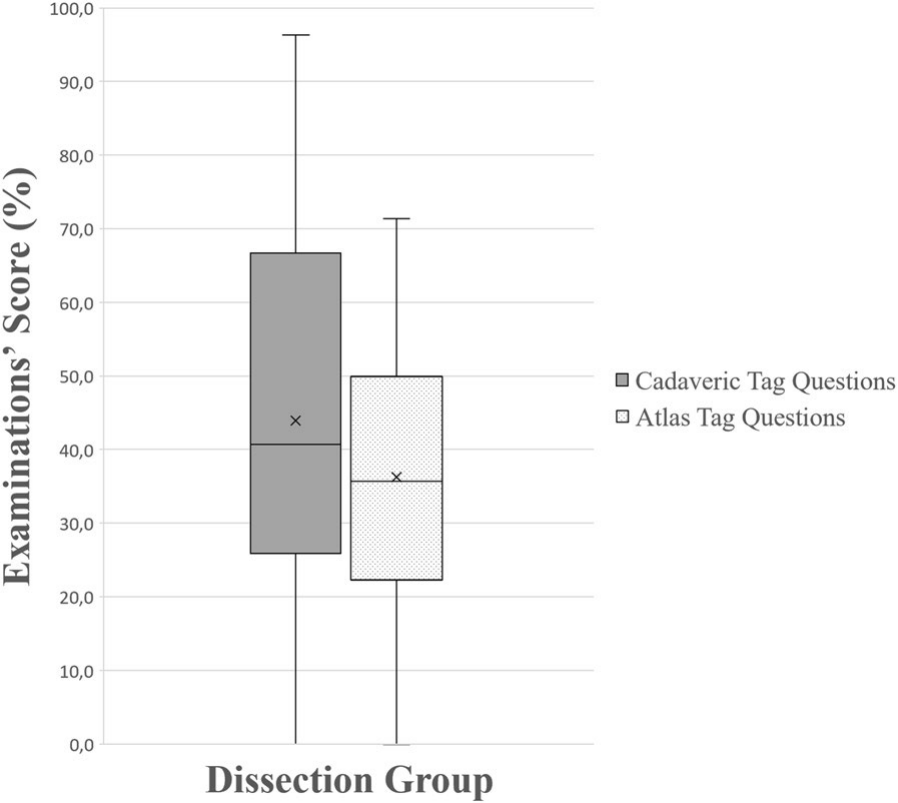
A boxplot of students’ scores (%) in the examinations, with whiskers from minimum to maximum. Performance of students using dissection is depicted for each question type (cadaveric tag questions and atlas’ tag questions). Segment inside the boxplot shows the median and “x” shows the mean. Students performed statistically significantly better in tag questions using cadaveric images (*p* < 0.001).

**Figure 8:**
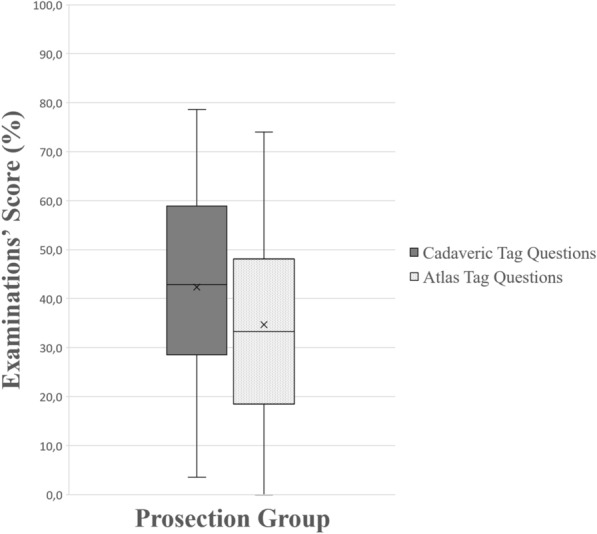
A boxplot of students’ scores (%) in the examinations, with whiskers from minimum to maximum. Performance of students using prosections is depicted for each question type (cadaveric tag questions and atlas’ tag questions). Segment inside the boxplot shows the median and “x” shows the mean. Students performed statistically significantly better in tag questions using cadaveric images (*p* < 0.001).

**Figure 9:**
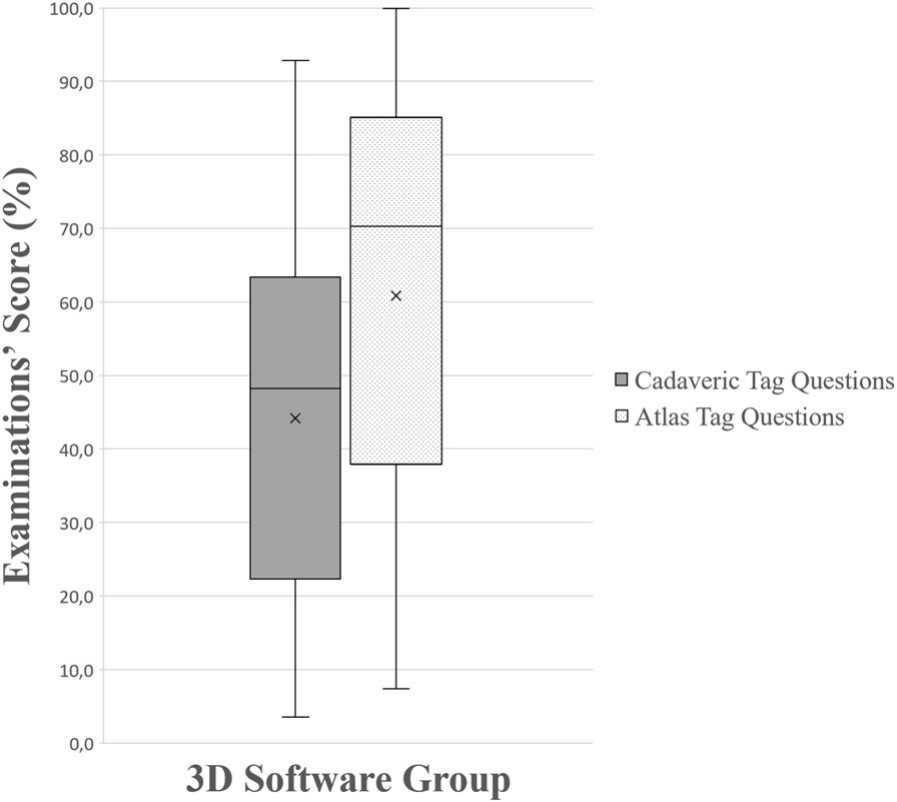
A boxplot of students’ scores (%) in the examinations, with whiskers from minimum to maximum. Performance of students using the 3D software is depicted for each question type (cadaveric tag questions and atlas’ tag questions). Segment inside the boxplot shows the median and “x” shows the mean. Students performed statistically significantly better in tag questions using atlas’ images (*p* < 0.001).

**Figure 10:**
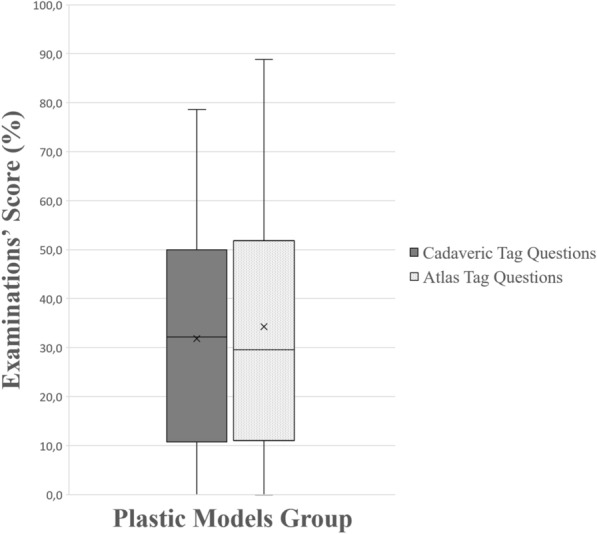
A boxplot of students’ scores (%) in the examinations, with whiskers from minimum to maximum. Performance of students using plastic models is depicted for each question type (cadaveric tag questions and atlas’ tag questions). Segment inside the boxplot shows the median and “x” shows the mean. Students performed equally in both types of questions (*p* = 0.42).

For each one of the four groups, performance of students in tag questions using cadaveric images and in tag questions using anatomy atlas’ images was compared. The dissection group performed statistically significantly better in tag questions using cadaveric images (*p* < 0.001), as it can be seen in Fig. 7. Similarly, the prosections group also performed statistically significantly better (*p* < 0.001) in tag questions using cadaveric images (Fig. 8). On the other hand, the 3D group performed statistically significantly better (*p* < 0.001) in tag questions using atlas’ images (Fig. 9), but students of the plastic models group performed equally in both types of questions (*p* = 0.42), as it can be seen in Fig. 10.

#### Gender-based analysis

No statistically significant difference between male and female students was observed, regarding performance in all types of questions in the dissection group, the prosections group, and the 3D group. However, in the plastic models group, female students outperformed male students in McQ (*p* = 0.01). Results can be seen in Table 3.

**Table 3:** Gender-based analysis using students’ t-test for all types of questions, in all four groups of the study.

Group	Question type	Mean score		Standard deviation (SD)		t-test (*p*)
		♂	♀	♂	♀	
Dissection	Total questions	47.1	52.8	20.7	19.2	0.26
	Tag questions	21.4	23.8	12.7	12	0.43
	Multiple-choice questions	25.7	29	8.6	8.2	0.12
Prosections	Total questions	38.5	42.8	17.4	17.4	0.28
	Tag questions	19.8	22.2	11.5	11.2	0.37
	Multiple-choice questions	18.7	20.6	6.9	7.5	0.22
Plastic models	Total questions	30.7	35.7	17.6	17.2	0.19
	Tag questions	16.2	17.8	12.7	12.3	0.56
	Multiple-choice questions	14.5	17.9	6.4	6.2	0.01*
3D software	Total questions	53.3	50.8	22.4	20.3	0.60
	Tag questions	28.7	27.8	14.9	12.6	0.78
	Multiple-choice questions	24.6	23	8	8.3	0.37

Bold value indicate statistical significance.

### Students’ perceptions of each learning modality (qualitative results)

After the end of the educational process, students evaluated the learning modality used during laboratory sessions. In total, 70% of all students were satisfied or very satisfied with their teaching modality. Additionally, 65.5% evaluated their modality as educational or very educational, and 69% as useful or very useful for their future clinical activities. However, 8% sensed severe fear or stress before the laboratory, a feeling which was also present in 4% of all students after the end of the educational process. Finally, 87% of all students stated that they would propose the course to next year’s students.

**Figure 11:**
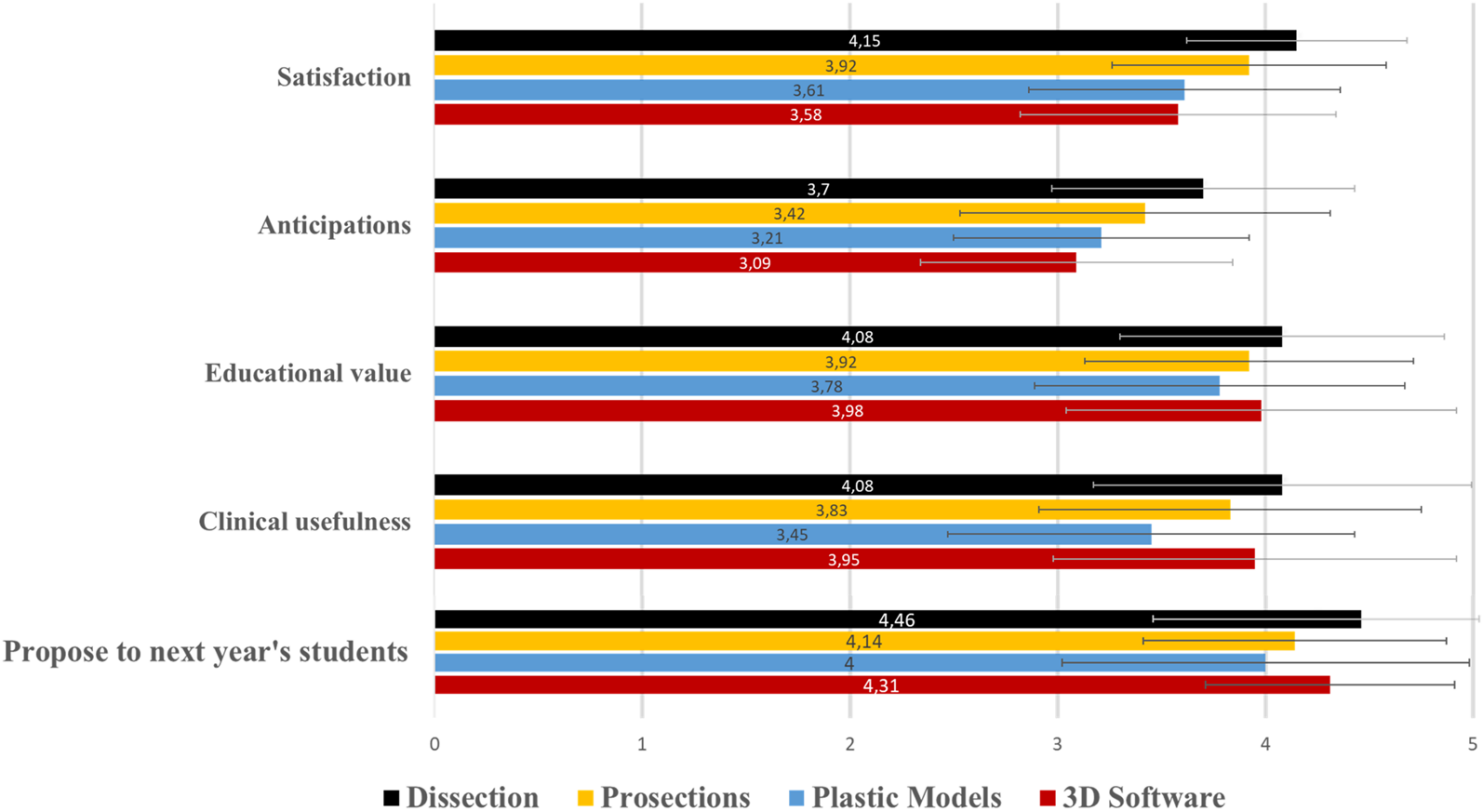
A histogram of students’ mean answers in Likert-scale questions evaluating the quality of the teaching modality and the participants’ intention to propose the course to the forthcoming students (1: min–5: max). Whiskers represent standard deviation (SD).

**Figure 12:**
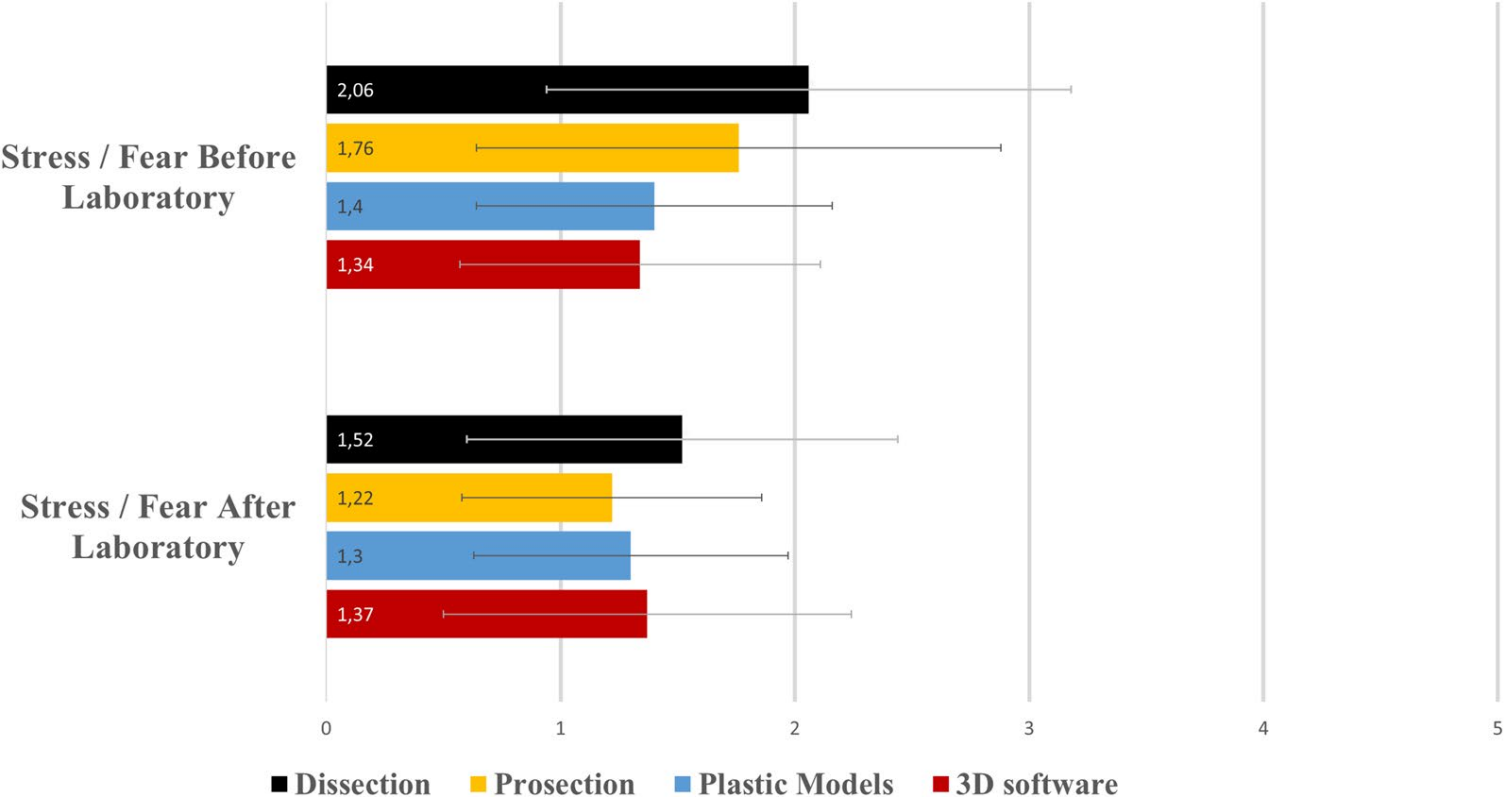
A histogram of students’ mean answers in Likert scale questions evaluating stress / fear before and after the laboratory sessions (1: min–5: max). Whiskers represent standard deviation (SD).

The null hypothesis of this study regarding students’ perceptions was also rejected since statistically significant differences in students’ evaluation were observed, as it can be seen in Table 4. Mean answers of each group in all questions are presented in Figs. 11 and 12. Responses were quantified for optical marking (e.g., 1: Very bad, 2: Bad, 3: Neutral, 4: Good, 5: Very good).

**Table 4:** Comparison of students’ perceptions using Kruskal–Wallis H test and Dunn’s post-hoc pairwise comparisons.

Questions	Kruskal–Wallis H test		Mean ranks				Dunn’s post-hoc comparisons
	***p***	H (3)	Dissection	Prosections	Plastic models	3D software	
Satisfaction	***p < 0.001***	33.118	195.08	166.79	137.81	126.61	1 > 3,4 and 2 > 4
Anticipations	***p < 0.001***	26.025	192.88	162.38	142.25	128.60	1 > 3,4
Educational value	*p* = 0.12	5.790	171.22	153.18	140.96	163.99	–
Clinical usefulness	***p < 0.001***	20.977	180.66	156.61	122.94	170.87	1,4 > 3
Fear/stress before	***p < 0.001***	26.367	189.87	164.51	139.31	133.08	1 > 3,4
Fear/stress after	*p* = 0.08	6.509	171.36	145.75	156.39	153.78	–
Propose to next year students	***p = 0.007***	12.162	180.84	145.30	140.08	162.76	1 > 2,3

Bold value indicate statistical significance.

Dissection received higher rates in all questions compared to the other three modalities, except for the question examining educational value, in which no significant difference (p = 0.12) between the four modalities was observed (Fig. 11). Higher rates in questions assessing satisfaction and anticipations were observed in the dissection group compared to the plastic models group (*p* < 0.001, *p* < 0.001) and the 3D software group (*p* < 0.001, *p* < 0.001). And, in the question evaluating clinical usefulness, dissection and 3D software received statistically significantly higher rates compared to plastic models (*p* < 0.001 and *p* = 0.003, respectively). In the questions evaluating students’ feelings, statistically significant differences were only found when students were asked for the time interval before the laboratory. Dissection stressed students significantly more compared to the use of plastic models and 3D software (*p* < 0.001, *p* < 0.001), whereas no difference was found when compared to prosection (*p* = 0.24), as it can be seen in Fig. 12. When examining stress/fear in the same group, before and after the laboratory sessions, differences were only observed in the dissection and the prosections group. However, even in this case, the percentage of students who continued to sense stress/ fear after the laboratory (5%) in the dissection group was statistically significantly lower compared to the percentage of students who sensed stress/ fear before the laboratory (13%; z-test comparison: *p* = 0.002), and the same was also observed in the prosection group (12% vs. 1%; z-test comparison: *p* = 0.008). Finally, the dissection group outperformed the prosection and the plastic models group in the question regarding proposing the course to the forthcoming students (*p* = 0.04 and *p* = 0.009 respectively).

In open-ended questions, students’ answers reflected the previously described statistical data. The vast majority of students (92%) asked for more time to study in the laboratory and stated that they would like to try one of the other learning modalities (85%). More specifically, students of the dissection and the prosection group stated that they would like to try the 3D software (72% and 80% respectively). On the other hand, students using the 3D software and plastic models asked for cadaveric specimens (76% and 78% respectively). Interestingly, 44% of the students of the dissection group considered their experience as the beginning of a surgical career and 60% of the students of the 3D group stated that they will use the software during studying anatomy in the forthcoming semesters, reflecting on the clinical usefulness of the two modalities. The most notable comments were made by students of the dissection group, characterizing their first dissection laboratory as a “unique”, “unbelievable” or “astonishing” experience. In fact, two students also brought up the humanistic care and end-of-life issues, usually encountered during cadaveric laboratories. The first one stated: “questions about life and death filled our daily discussions for days, thank you for making us feel like doctors for the first time”. The second one stated: “Something fascinating happened today since I met my first patient. It’s called medicine and it will last forever”. Nevertheless, negative comments were also present by a small percentage of those using cadaveric specimens. Two students characterized dissection as a “disgusting experience” and a female student stated that she does not want to participate again in a same laboratory. Finally, one disappointed student from the 3D group stated that he was frustrated to perform his first dissection on a laptop screen.

## Discussion

Summarizing the results, it is rather clear that there are differences between the four modalities, reflected both in students’ performance and in students’ perceptions. To our knowledge, this is the first study comparing dissection, prosections, plastic models and 3D software simultaneously, in teaching and learning musculoskeletal anatomy.

Data analysis showed that dissection and 3D software are more effective than prosections and plastic models when targeting gross anatomy knowledge acquisition. In fact, when comparing these two modalities, the sub-analysis provided even more interesting results: the 3D group performed significantly better in tag questions and the dissection group performed significantly better in McQ. The superiority of the BioDigital Human software in tag questions reflects on its ability to enhance spatial anatomy acquisition. Isolation and 360° view for each anatomical structure are not available during dissection and this is maybe the main advantage of the contemporary 3D anatomy software over dissection. On the other hand, dissection offers a touch-mediated perception of the human body which cannot be simulated, enhancing the acquisition of more complex information^31^. And maybe, that is why a statistically significant superiority of dissection compared to the 3D software was observed in Bloom’s level 2 McQ. Regarding literature, there is only one study comparing these two modalities. Codd et al. found no difference between their 3D software and dissection, using 10 questions, in a small sample of 36 students, including a control group^32^. Their study targeted forearm anatomy, they did not use a commercially available software and their sample was too small, but their findings were consistent with our results since in total questions no statistically significant difference between dissection and 3D software was observed in the present study too. Although there are no other studies comparing 3D software to dissection, Hisley et.al. examined another 3D visualization technology^33^. They reported superiority of 3D MRI–CT compared to dissection, in questions examining spatial knowledge acquisition, although their sample was again small (only 16 students). These results also comply with the findings of the present study, since in tag questions, students of the 3D group performed significantly better compared to those of the dissection group, highlighting once again the importance of isolation and 360° view of each structure provided by contemporary 3D software.

Dissection is not only effective but also first in students’ preferences. In the present study, dissection received statistically significantly higher rates in questions evaluating students’ satisfaction and anticipations compared to plastic models and 3D software. Dissection was also the only modality characterized as a “unique”, “unbelievable” or “astonishing” experience by students in open-ended questions. The same results have been reported by many previous studies, which shows that despite recent changes in teaching, dissection remains the gold standard in anatomy learning for students^34–37^. However, no differences were found between dissection and the BioDigital Human software in students’ perceptions regarding clinical value. This is interesting because both modalities are considered useful for students’ future clinical activities, but for different reasons. Dissection enhances the development of practical skills, mainly because of the use of basic surgical instruments during the procedure. That is why 44% of the students of the dissection group considered these laboratories as the beginning of their surgical careers, which has been also been reported by other studies^34,38^. On the other hand, 60% of the students of the 3D software group stated that they would definitively choose to use a 3D software during studying anatomy for future clinical activities. It is rather clear that the 3D software is the only modality that can be easily used at home, during self-study and so it is considered clinically useful in a vertically integrated medical curriculum.

As far as prosections are considered, satisfaction and anticipations rates were somewhat lower but without statistical significance compared to dissection. Indeed, pre-dissected specimens (prosections) are regarded not only as a valuable alternative to dissection but also as a time-saving modality, increasing teaching time^8,31,35,36,39–42^. In a recent review comparing dissection to prosections, a marginal superiority of dissection was reported by Winkelman et al.^43^. Similarly, when focusing on musculoskeletal anatomy teaching, results are controversial. Sinclair et al. reported a marginal superiority of dissection in tag and McQ, in a sample of 219 students studying anatomy of the lower limb, but they found no difference in open-ended questions^39^. In an even bigger sample of about 500 students, Jones et al. compared dissection to prosections in head and neck, upper and lower limb anatomy. No difference was found in 26 out of the 35 examination tests, dissection outperformed prosection in 3 tests and prosection in 6 tests^44^. Moreover, Peppler et al. reported no difference in a sample of 30 students studying upper and lower limb anatomy, but in a similar study with a slightly bigger sample, 5 years later he reported superiority of dissection in 5 of the 24 tests^45,46^. On the other hand, Nnodim et al. reported superiority of prosections in a well-structured study, examining anatomy learning of the lower limb and in the most recent study, Peeler et al. found no significant difference between dissection and prosection, used in a modern curriculum and integrated with 3D software, clinical cases, and radiology examinations^40,42^. Although literature review is controversial, in the present study a clear superiority of dissection over prosection was observed in total and McQ. However, in tag questions no significant differences between the two modalities were observed. This reflects on the opinion that prosections may be valuable in learning basic structures during studying gross anatomy, but when it comes to more complex information, like the exact structure’s route, dissection seems more effective. Additionally, superficial structures e.g. nerves and more delicate structures like synovial sheaths are destroyed during preparation or subjected to major changes regarding texture and color, and thus, they should not be taught using prosections^41,47,48^. Considering the serious lack of cadavers, in Europe and in the rest of the world, prosections are surely a significant alternative when it comes to cadaveric laboratories. However, they should not replace dissection, but on the contrary, their targeted use could be beneficial, saving both teaching time and cadavers^6,7^.

Interestingly, there are is only one study comparing 3D software to prosections. Mitrousias et al. reported superiority of the 3D software compared to prosections in a similar study^21^. Their results, observed in a smaller sample (72 students), comply with the findings of the present study. There is also one study comparing a CT-based, 3D anatomy model with prosections, in anatomy of the muscles of mastication. Hopkins et al. tested this model in 74 students and found no significant difference in performance between the group using prosections, the group using the 3D model, and the group using both modalities^49^. However, it should be highlighted that they used a 3D model, not a commercially available software. In our study, the group using prosections had statistically significantly lower examination scores compared to the 3D group in all types of questions. Learning by doing is less present when using prosections. This fact along with the convenience of using a 3D software (isolation of structures, accompanying information of origin, insertion, nerve and blood supply) may explain the superiority of the BioDigital Human software, compared to prosections. However, students’ satisfaction rates were higher in the prosection group compared to the 3D group, emphasizing once again the strong desire of medical students to study on cadaveric specimens during anatomy, in the medical curriculum.

Finally, the lower scores achieved by the group using plastic models were statistically significant when compared to all other groups in total and McQ, and also in tag questions when compared to the 3D group. Once again, there are only two studies comparing plastic models to another teaching modality. Khot et al. compared plastic models of the pelvis to 2D images and a 3D-reconstruction of a CT scan, reporting a superiority of plastic models in tag questions^50^. These results cannot be compared to the present study’s findings, since Khot et al. did not use a commercially available software but instead, a CT, 3D reconstruction. In the second study, Mitrousias et al. found no difference in students’ scores when comparing plastic models to prosections, in a smaller sample of 60 students in total^23^. Indeed, plastic models are considered as useful memory aids, corresponding to the human body in the spatial relationships of the represented structures and enhancing three-dimensional comprehension and anatomical reasoning^9^. In fact, a recent meta-analysis showed their effectiveness, especially in spatial knowledge acquisition and long-retention knowledge but it should be noted that they are usually low-fidelity copies, representing only a small number of the existing structures of a specific region and sometimes not accurately regarding shape and surface details^9,10^. These findings comply with the results of the present study since the group using plastic models performed worse compared to other groups in total and McQ, but with no significant difference compared to the dissection and the prosections group regarding tag questions.

Moreover, the question-based analysis showed interesting results. Students of the dissection group performed better in tag questions using cadaveric images. Similarly, students of the prosection group also performed better in tag questions using cadaveric images. On the other hand, students of the 3D group performed better in tag questions using atlas’ images. However, students of the plastic models group performed equally in both types of questions. Thus, lack of familiarization with cadaveric images seemed to be no problem for the plastic models group. This observation supports “the transfer of learning” theory, namely the extent to which knowledge learned in one context is applied in another^51,52^. Students performing dissection or using prosections were unable to transfer their knowledge to atlas’ images. Similarly, students using the 3D software had difficulty transferring their knowledge to cadaveric images. On the contrary, students using plastic models were favored to the extent that they were able to transfer their knowledge from plastic models (one context) to cadaveric images (second context). This may mean that plastic models could be successfully used as an introductive modality for cadaveric laboratories, resembling the true dimensions of the human body and providing a rather similar to cadaveric specimens, 3D, touch-mediated perception of the human body, a finding also reported in previous studies^23^.

As far as students’ perceptions are considered, plastic models were evaluated significantly lower in questions regarding satisfaction, anticipations and clinical usefulness compared to dissection. However, no difference was observed between the four groups in the question regarding educational value of the modality. It should be stated once again that plastic models cannot replace dissection or prosections. This opinion is clearly reflected in students’ answers. Although plastic models are considered important when targeting spatial knowledge acquisition, they lack other important characteristics. Anatomic variability seen during dissection and in prosections cannot be observed when using plastic models. And of course, the same is also true for the development of practical skills, which should be kept in mind when developing future anatomy curricula. Lastly, the only significant difference in gender-based analysis was found in the plastic models group, where female students outperformed male students in McQ, which is merely a random finding with no rational explanation.

The evaluation process did also reveal a few more covert learning outcomes. First of all, the majority of students asked for more time in the laboratory, highlighting the need to increase teaching hours in anatomy, which are constantly decreasing as it has been thoroughly reported^3–5,53,54^. The majority of students also asked to try one of the other teaching modalities, which implies that a combination of methods, already requested in many studies, could be used to achieve different educational goals and ameliorate anatomy knowledge^32,34,37,39,49^. Secondly, a few students were severely annoyed by dissection, such as the male student characterizing the process disgusting or the female student stating that she would not like to participate again in a similar laboratory. This indicates that dissection may not be appropriate for some, and their personality traits may play a significant role in this decision^55^. However, in general, the initial stress of students performing dissection and using prosections was significantly reduced after laboratories. This reduction may be attributed to familiarization with the human body and corresponding emotional preparation, which is a main aspect of the “hidden curriculum” that only cadaveric preparations can provide to students. Introduction to humanistic care, doctor-to-patient respect and end-of-life issues, can be discussed during cadaveric laboratories, which eventually reduces students’ fear, acting as a preparation for their clinical duties and promoting psychosocial development^31,56,57^. Unfortunately, such discussions are rarely induced during virtual dissection on 3D software, which is also reflected in the disappointment of one of the students of the 3D group. Although in a recent meta-analysis comparing laboratory instructional approaches in high school, physiotherapy, veterinary and medical students, no difference was found between dissection, prosection, plastic models, and digital media in the context of short-term knowledge gain, additional educational objectives like those mentioned above should be taken into consideration since they are not fulfilled when using certain modalities, like plastic models or 3D software^58^. This is important for all learners, but especially for medical students, due to the special characteristics of their daily practice.

### Limitations of the study

In this study, students used only one learning modality. So, it is not possible to safely conclude which one is the most preferable one. Although the educational time frame was strictly the same for all groups, students may have used alternative learning materials on their own. However, they were clearly advised not to and it is also difficult for first-year medical students to familiarize and use additional learning resources (e.g. a different book or atlas) in three weeks.

Additionally, although the study’s timeframe corresponded to the official timeframe of the medical curriculum of the host university, laboratory time was limited. For example, time for dissection was 4 h for the whole upper limb, making the whole process even more demanding for novice students. Thus, results may not be applicable to the way anatomy is taught in other institutions or other countries. However, efforts were made to take full advantage of the laboratory hours and in this way, removal of skin and subcutaneous tissue was performed in all fresh-frozen cadaveric specimens before used by students.

Lastly, these are results from only one department, targeting a specific anatomical region, using specific models and specimens, and only one of the commercially available 3D software, as described in the materials and methods section. They should not be generalized since different software or models of different quality may result in different findings. Also, this study’s findings cannot be applied in all types of anatomy learning (cross-sectional anatomy, surface anatomy, etc.).

## Conclusions

The majority of medical schools currently use a combination of the above teaching modalities^59^. The conclusion of this article aims to put each modality in the right educational context and promote the appropriate and targeted use of all four modalities in anatomy teaching and learning. Dissection remains the gold standard modality, a fact which is reflected both in examination scores and in students’ perceptions. It is sure that the use of cadaveric specimens is expensive, requires time and facilities, and may involve various risks. However, educators should continue placing their trust in this modality, since a targeted and methodical use of dissection could maximize its potentials. Prosections are a valuable alternative. They cannot replace dissection, but their use could be a solution to problems like shortage of cadavers and lack of teaching time. Their contribution in teaching gross anatomy is proven. Three-dimensional anatomy software are an emerging teaching modality. They should not replace cadaveric specimens. Instead, they should be used in combination with them since their contribution in spatial anatomy knowledge acquisition is undeniable. Easy to use, cheap, available at home and constantly developing, 3D software is the future of anatomy learning. Finally, plastic models should be also included in anatomy teaching. Available and popular in many departments, they could be successfully used as memory-aids and introduce students to basic anatomy elements, especially when preparing for cadaveric laboratories. Low-fidelity issues may arise and high-quality should be a priority. As a take-home message, all available learning modalities should be used. A carefully planned combination could help tutors achieve more educational goals.

## Supplementary information

Supplementary information.
