# Supplementary material for: Musculoskeletal anatomy: evaluation and comparison of common teaching and learning modalities
Source: Sci Rep. 2021 Jan 15;11:1517. doi: 10.1038/s41598-020-80860-7 (PMC7810993; doi:10.1038/s41598-020-80860-7)
Supplement: Supplementary file 1 — Supplementary information. [file 41598_2020_80860_MOESM1_ESM.docx]

**Appendix**

***1. Example of tag questions using cadaveric images.***


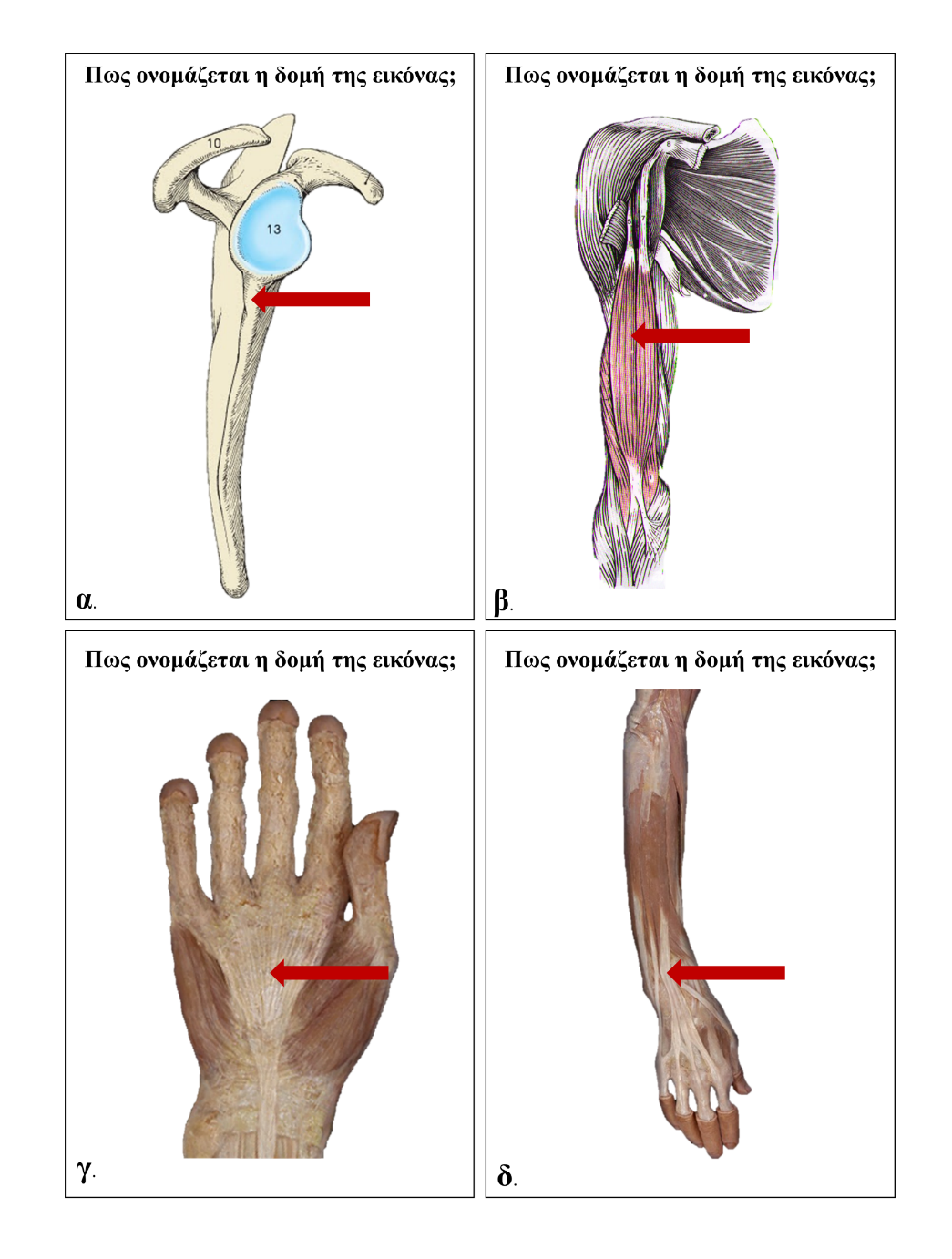


**Name the structure pointed**

**by the red arrow.**

**Name the structure pointed**

**by the red arrow.**

***2. Example of tag questions using atlas’ images.***

**
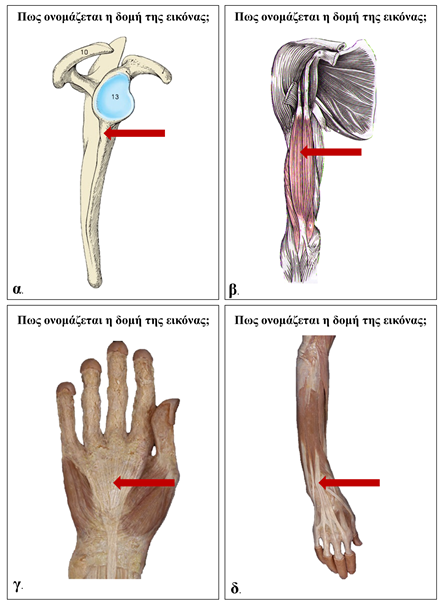
**

**Name the structure pointed**

**by the red arrow.**

**Name the structure pointed**

**by the red arrow.**

***3. Example of multiple-choice question (bloom’s level 1).***

The supraspinatus tendon is inserted into:

a) the lesser tubercle

*b)* ***the superior facet of the greater tubercle***

c) the supraglenoid tubercle

d) the inferior facet of the greater tubercle

***4. Example of multiple-choice question (bloom’s level 2).***

Which of the following structures pass through the Guyon’s canal?

a) median nerve and radial artery

***b) ulnar nerve and ulnar artery***

c) radial nerve and radial artery

d) deep branch of the radial nerve and ulnar vein

***5. The questionnaire used during the evaluation process.***

| **Question** | **Part** |
| --- | --- |
| Sex | I |
| Age | I |
| **How satisfied you are from the teaching modality you used?**  (1: very dissatisfied, 2: dissatisfied, 3: neutral, 4: satisfied, 5: very satisfied) | II |
| **Regarding your anticipations, the teaching modality you used was:**  1: much worse than anticipated, 2: worse than anticipated, 3: matched anticipations, 4: better than anticipated, 5: much better than anticipated. | II |
| **In your opinion, how educational is the teaching modality you used?**  (1: not at all educational, 2: slightly educational, 3: neutral, 4: educational,  5: very educational) | II |
| **In your opinion, how useful is the teaching modality you used,**  **for your future clinical skills?**  (1: not useful at all, 2: slightly useful, 3: neutral, 4: useful, 5: very useful) | II |
| **Did you feel any stress / fear before the laboratory?**  (1: not at all, 2: not really, 3: somewhat, 4: much 5: very much) | II |
| **Did you continue to feel stress / fear after the laboratory?**  (1: not at all, 2: not really, 3: somewhat, 4: much 5: very much) | II |
| **Would you propose to the forthcoming students to participate in the course?**  (1: surely no, 2: no, 3: maybe, 4: yes, 5: surely yes) | III |
| **Comments. What would you like to be different / to be added in the educational process?** | III |
